# Neural Responses in the Primary Auditory Cortex of Freely Behaving Cats While Discriminating Fast and Slow Click-Trains

**DOI:** 10.1371/journal.pone.0025895

**Published:** 2011-10-05

**Authors:** Chao Dong, Ling Qin, Yongchun Liu, Xinan Zhang, Yu Sato

**Affiliations:** 1 Department of Physiology, Interdisciplinary Graduate School of Medicine and Engineering, University of Yamanashi, Chuo, Yamanashi, Japan; 2 Clinical Medicine Research Center, Inner Mongolia Medical College, Huhhot, People's Republic of China; 3 Department of Physiology, China Medical University, Shenyang, People's Republic of China; University of New South Wales, Australia

## Abstract

Repeated acoustic events are ubiquitous temporal features of natural sounds. To reveal the neural representation of the sound repetition rate, a number of electrophysiological studies have been conducted on various mammals and it has been proposed that both the spike-time and firing rate of primary auditory cortex (A1) neurons encode the repetition rate. However, previous studies rarely examined how the experimental animals perceive the difference in the sound repetition rate, and a caveat to these experiments is that they compared physiological data obtained from animals with psychophysical data obtained from humans. In this study, for the first time, we directly investigated acoustic perception and the underlying neural mechanisms in the same experimental animal by examining spike activities in the A1 of free-moving cats while performing a Go/No-go task to discriminate the click-trains at different repetition rates (12.5–200 Hz). As reported by previous studies on passively listening animals, A1 neurons showed both synchronized and non-synchronized responses to the click-trains. We further found that the neural performance estimated from the precise temporal information of synchronized units was good enough to distinguish all 16.7–200 Hz from the 12.5 Hz repetition rate; however, the cats showed declining behavioral performance with the decrease of the target repetition rate, indicating an increase of difficulty in discriminating two slower click-trains. Such behavioral performance was well explained by the firing rate of some synchronized and non-synchronized units. Trial-by-trial analysis indicated that A1 activity was not affected by the cat's judgment of behavioral response. Our results suggest that the main function of A1 is to effectively represent temporal signals using both spike timing and firing rate, while the cats may read out the rate-coding information to perform the task in this experiment.

## Introduction

Biologically relevant sounds, such as speech, animal vocalizations and music, contain a quasi-periodical repetition of acoustic events [1], [2]. For human listeners, auditory perception varies with the repetition rate of an acoustic event. Low repetitions (from 10 Hz to ∼45 Hz) are heard as distinct fluctuations in loudness known as acoustic flutter, whereas higher repetition rates (50 up to ∼300 Hz) are heard increasingly as rough continuous sound [3], [4]. To reveal the neural encoding of temporal signals, a number of electrophysiological studies have been conducted on various animals. A common finding is that the majority of auditory cortex neurons exhibited a sequence of transient discharges synchronized with each acoustic event in the repeated stimuli, and the upper limit of synchronous response is about 30 Hz with some variation depending on the anesthetic and the method of measuring response synchrony [5], [6], [7], [8], [9], [10], [11], [12], [13], [14], [15], [16]. It has been proposed that precise spike timing may code slow repetition sounds, while faster repetition sounds may be encoded by the neural firing rate during the entire stimulus period [3], [11], [15], [17]. This possibility was highlighted by recent experiments on awake marmosets [18], [19], [20], which found that some cortical neurons show sustained discharge throughout the stimulus duration, resulting in a mean firing rate proportional to the stimulus repetition rate. Non-synchronized rate-coding of temporal signals has also appeared in papers on awake macaques [12], [21], while the percentage of non-synchronized response largely varied among different studies because of the many differences in the experimental factors; nevertheless, the current data support that auditory cortical neurons may use multiple features to robustly represent sound repetition rates.

One of the key questions in sensory neuroscience concerns which features of the neural code are “read out” to support perception. An effective approach to address this question is to carry out behavioral testing of perceptual performance and electrophysiological recording in parallel. Such methodology has long been used in visual and somatosensory neuroscience [22], [23]; however, it has rarely been adopted to investigate how much a neural response might contribute to an animal's perception of repetition sounds on a trial-by-trial basis. To date, only one attempt has been made by Lemus et al. to record neural responses in the primary auditory cortex (A1) when monkeys were discriminating the frequency difference between two sequentially presented acoustic flutters, in which the repetition rate varied in the range of 14–40 Hz [24]. They found that the monkeys' psychophysical discrimination of acoustic flutters is matched better by neural discrimination estimated from the firing rate than that from the firing periodicity. They therefore suggested that the firing rate, and not spike timing, represents the neural code that at the level of A1 is relevant for acoustic flutter discrimination. Because Lemus et al. only examined low repetition rates and neural activity may vary among species and behavioral contexts, far more experimental data are needed to draw a common conclusion on the neural basis of repetition sound perception.

For this reason, we trained cats to discriminate click-trains at low and high repetition rates using an established Go/No-go procedure, and simultaneously recorded the single-unit activities in A1 through the implanted electrodes. By comparing the cats' behavioral performance with recorded neural activities, we attempted to address how evoked neuronal activity in A1 is read out to direct the cat's behavioral responses during click-train discrimination.

## Results

### Psychophysics

**Figure 1:**
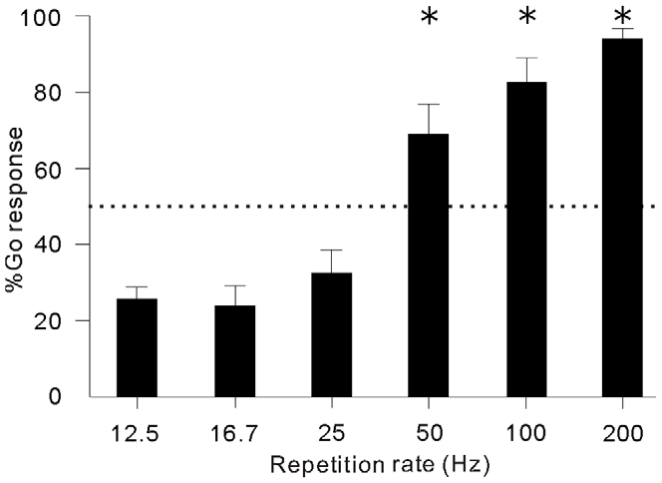
Percentages of Go responses in cats hearing click-trains at different repetition rates. Height of a black bar represents the mean % Go averaged over the sessions of 7 cats. Short horizontal line represents 2SE. Dotted line shows the 50% Go response. Asterisk marks the statistical significance of p<0.01(ANOVA followed by a Tukey-Kramer multi-comparison procedure).

We first trained seven cats (5 males and 2 females) to discriminate periodic click-trains at 12.5 and 200 Hz repetition rates using a Go/No-go procedure, wherein cats were rewarded for licking a metal pipe after the presentation of a 200 Hz click-train, and not rewarded after the presentation of a 12.5 Hz click-train (see Materials and Methods). After 3, 000–4, 000 trials of behavioral training, all cats learned this acoustic task and achieved high performance (d'≥2.0). We then evaluated the cats' responses to randomly presented click-trains at 6 different rates (12.5, 16.7, 25, 50, 100, 200 Hz) in one session. The mean and 2× standard error (SE) of the percentage of Go responses (%Go) was plotted as a histogram against the repetition rate (Fig. 1). The 7 cats showed a high percentage of Go responses (>50%) to click-trains at 200, 100 and 50 Hz repetition rates, while a low percentage to 25, 16.7 and 12.5 Hz stimuli. Statistical analysis (ANOVA followed by a Tukey-Kramer multi-comparison procedure) confirmed that %Go of 50, 100 and 200 Hz was significantly higher than that of 12.5 Hz (p<0.01). This result indicates that the cats' ability to distinguish 200 Hz from 12.5 Hz click-trains was only generalized to correctly discriminate the difference between 50 and 12.5 Hz repetition rates.

### Neural responses of individual units

To reveal the neural substrate of auditory perception, we recorded spike activities from 92 sites in both hemispheres of the caudal part of A1 (characteristic frequency ranged from 0.5 to 16 kHz), as the subjects were performing the auditory task. A1 neurons responding to the click-trains showed a variety of temporal firing patterns. Several representative examples of the neural responses are present as follows.

**Figure 2:**
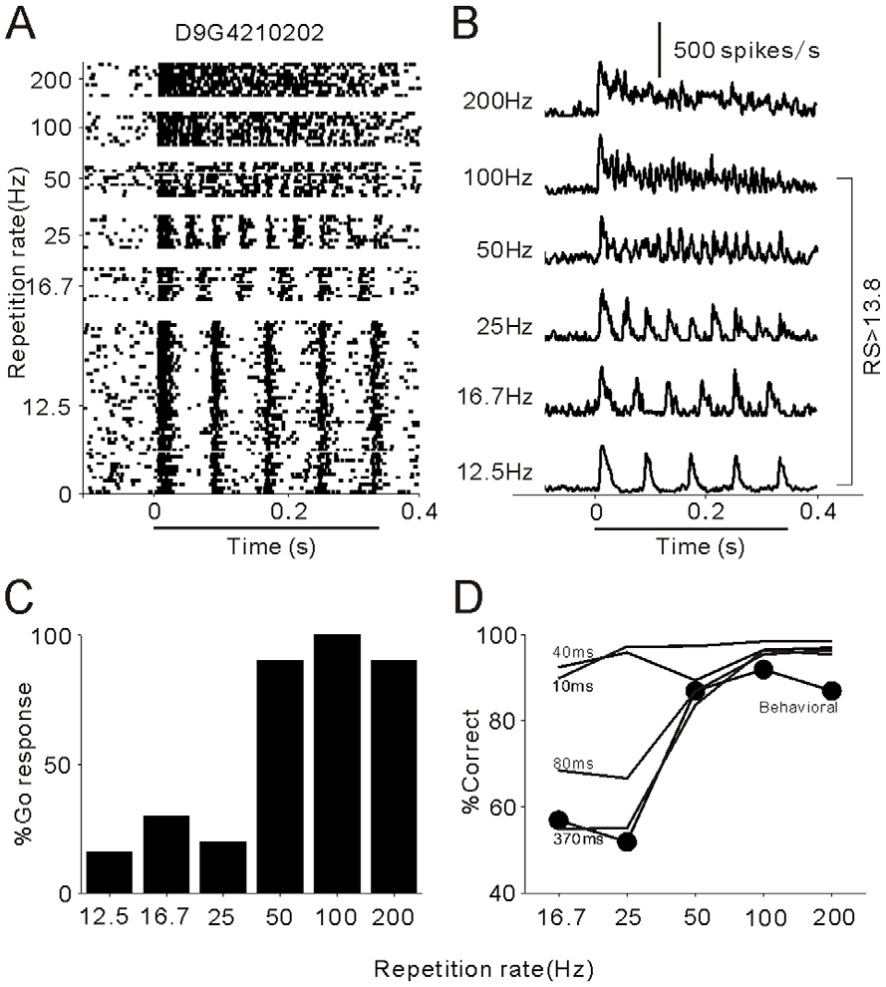
An example of spike activities of A1 units when the cat was discriminating the click-trains. (A) Raster plots of neural spikes in 50 trials of standard stimuli (12.5 Hz) and 10 trials of target stimuli (16.7–200 Hz). Stimulus duration is indicated by the horizontal bar below the time axis. (B) PSTH representing the mean firing rate (1-ms bin, smoothed by Gaussian function with 5-ms SD) in each stimulus condition. (C) Cat's behavioral response (% Go) during the unit recording. (D) Psychometric and neurometric functions. Filled circle marked line represents the psychometric function. Thin lines represent the neurometric functions calculated from this neuron based on different temporal resolutions.

Figure 2A and 2B present the spike activities of an example A1 unit responding to click-trains at different repetition rates. At 12.5 Hz, the timings of the clicks were explicitly represented by the timings of the spikes, with little adaptation in the strength of the spike response. Such a phase locked response pattern remained when the click repetition rate increased from 12.5 to 100 Hz, but disappeared at the click repetition rate of 200 Hz. These observations were evaluated by Rayleigh Statistic (RS), which showing the statistical significance of spike synchronization to clicks (See Materials and Methods). RS in this unit was higher than 13.8 (corresponding to p <0.001 in the Rayleigh test) at 12.5–100 Hz repetition rates, and lower than 13.8 at 200 Hz, indicating that the high cutoff of the synchronized response was 100 Hz in this unit. Also, the mean firing rate of this unit monotonically increased with the increase of the repetition rate. Statistical analysis (ANOVA) indicated that the change in the mean firing rate was significant (p<0.001).

Next, we determined whether the neural signal carries information in a way that may account for the animal's psychophysical behavior. Figure 2C presents the %Go response of this cat during unit recording, which showing a tendency similar to that observed in the previous psychophysical experiments (Fig. 1). To quantify the cat's performance in the discrimination of different repetition rates from 12.5 Hz, we adopted an “adjusted measure” of the proportion correct [25]: *p(correct)  =  [p(hits)+*(1−*p(false alarms)*)*]* / *2*. If the subject shows a Go response in all target trials [*p(hits) = 100%*], but not in any standard trials [*p(false alarms) = 0*], the % correct is 100%, indicating perfect discrimination. The % correct of discriminating each target (16.7, 25, 50, 100 and 200 Hz) from the standard (12.5 Hz) constructed the psychophysical function of repetition rate discrimination (filled circles in Fig. 2D).

We then computed neurometric functions based on the PSTHs at different temporal resolutions (10, 40, 80 and 370 ms, see Materials and Methods), which are illustrated by the lines in Fig. 2D, respectively. The PSTHs at 10 and 40 ms resolutions contain precise spike timing information, from which an ideal observer can correctly discriminate 12.5 Hz click-trains from all other stimuli (16.7–200 Hz). In contrast, the PSTH of 80 ms resolution captured more firing rate information but lost some spike time information. Consequently, the discrimination performance at 16.7 and 25 Hz (60 and 40 ms inter-click interval) deteriorated. The PSTH of 370 ms resolution actually contains only one bin corresponding to the mean firing rate across the period from stimulus onset to 50 ms after stimulus offset. The neurometric function based on the mean firing rate shows high performance at 50–200 Hz but low performance at 16.7–25 Hz, which is the best match of the cat's behavioral performance. This result suggests that the spike activity of this unit provides sufficient information to discriminate all the click-trains if it is read out in a short time scale (≤40 ms), but the cat may refer to spike activity in a long time scale (≥80 ms) to perform the task.

**Figure 3:**
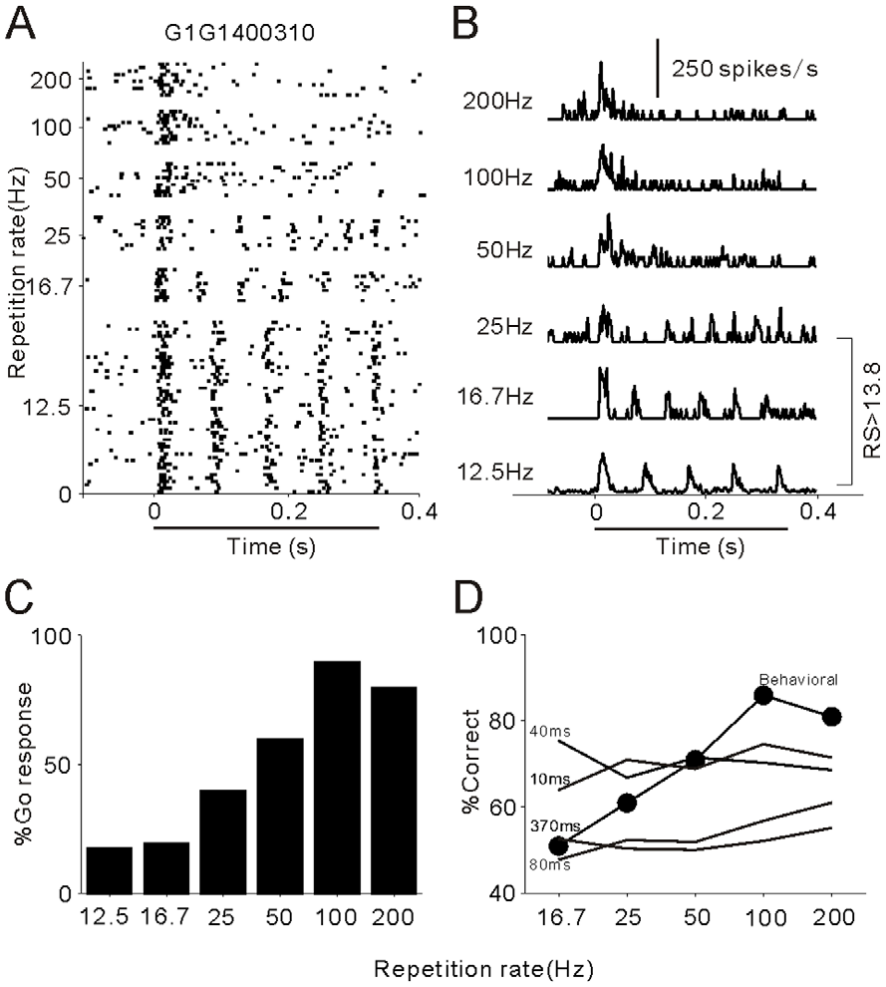
The other example of synchronized units with a lower synchrony cutoff (25 Hz). Same format as Fig. 2.

Figure 3A and 3B present the other unit with a lower ability to synchronize to the clicks. This unit showed precise stimulus-synchronized spikes to 12.5–25 Hz click-trains (RS > 13.8); however, for a repetition rate higher than 50 Hz, the response consisted only of an onset to the click-train followed by adaptation that lasted for the duration of the stimuli. Consequently, the high cutoff of the synchronized response was 25 Hz in this unit. Compared with the cat's behavioral response (Fig. 3C and D), the neurometric functions in shorter time scales (10–40 ms) showed a steady performance around 75% correct, obviously differing from the monotonically increasing psychometric function. While neurometric functions in longer time scales (80–370 ms) showed poor discrimination performance at any repetition rates, this was attributable to the mean firing rate of this unit not being significantly modulated by the change of the repetition rate (p>0.05, ANOVA). Thus, the subject's behavioral response may not directly correlate to the activity of this unit.

**Figure 4:**
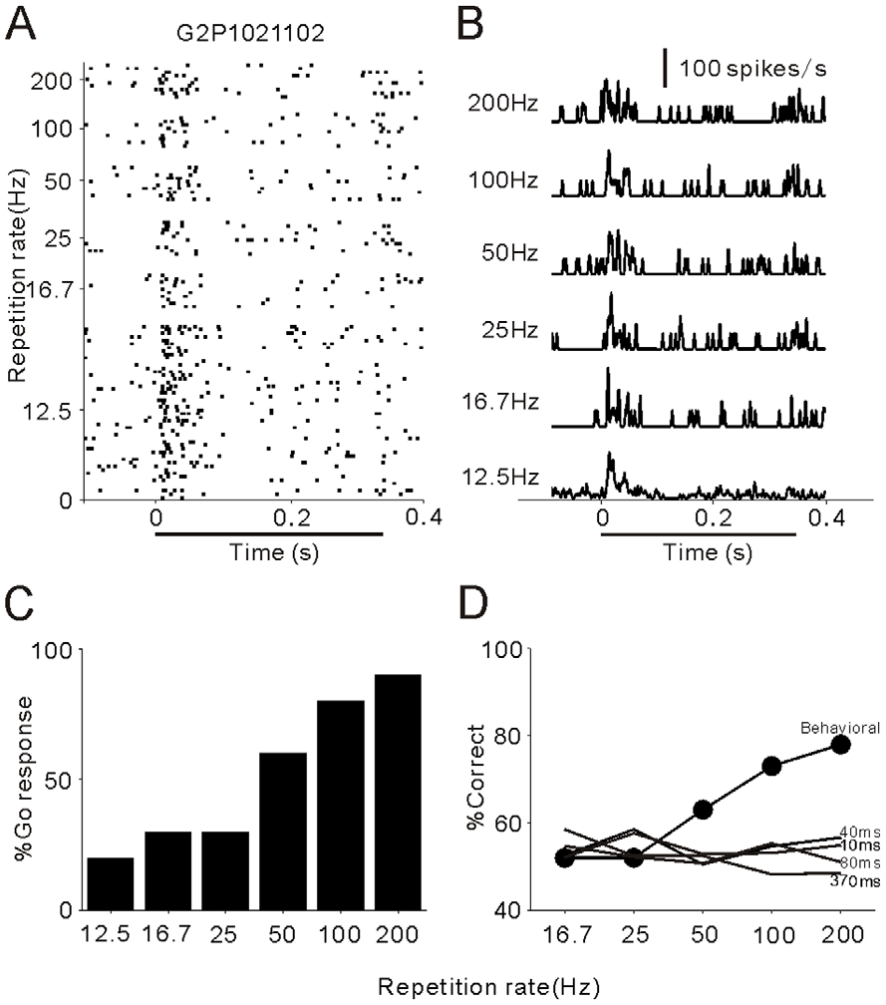
An example of non-synchronized units showing similar responses to the click-trains at all repetition rates. Same format as Fig. 2

We have presented above two examples of synchronized units which showed various degrees of synchronized responses to click stimuli. There were also some units, which had no ability to follow the click at any repetition rates; one example is presented in Fig. 4. No matter which repetition rate was applied, this unit showed a dominant response at the onset and a weak response at the offset of the entire click-train (Fig. 4A and B). RS was lower than 13.8 at any repetition rate, and the mean firing rate was not significantly modulated by the repetition rate (p>0.05, ANOVA). Thus, neither the spike time nor firing rate of this onset-response unit can provide useful information for discrimination of the repetition rate (Fig. 4C and D).

**Figure 5:**
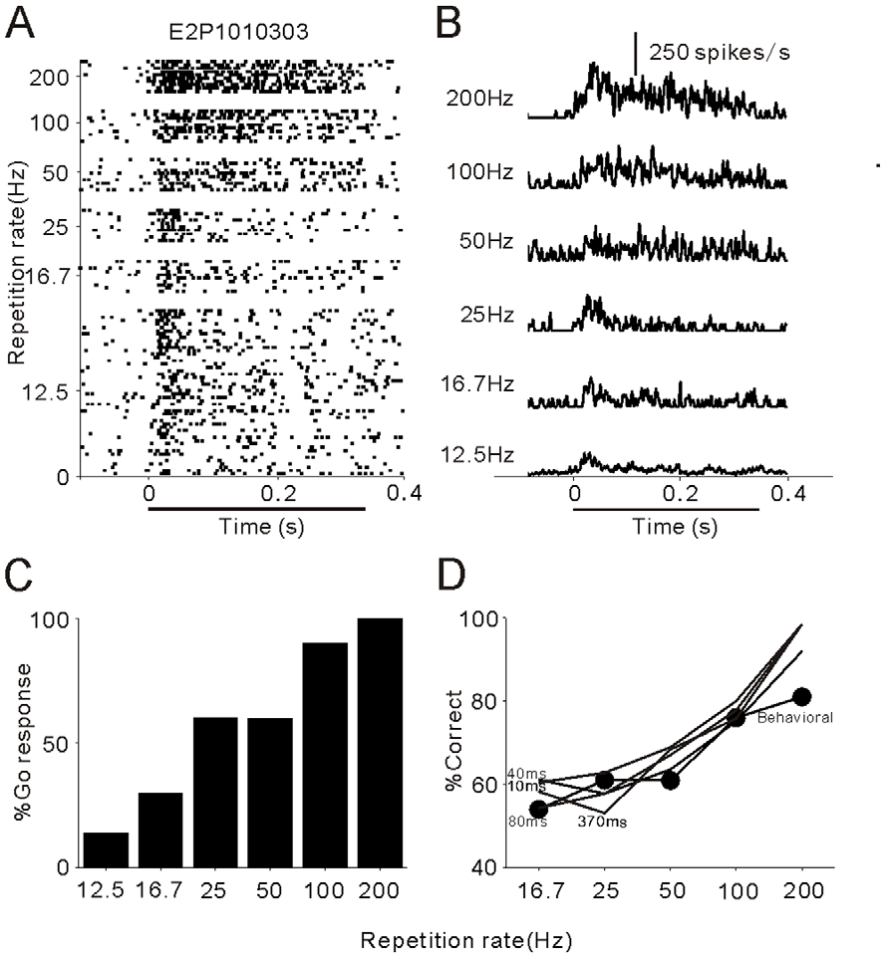
An example of non-synchronized units showing a monotonic increase in firing rate with the increase of stimulus repetition rate. Same format as Fig. 2

Interestingly, we also found a few units, in which the mean firing rate gradually increased with the increase of the repetition rate (Fig. 5A and B). The change in the mean firing rate was statistically significant (p<0.001). This kind of response pattern corresponds to the non-synchronized unit reported in the study on awake marmoset (Fig. 2a in [20]). The neurometric function based on the mean firing rate (370 ms time window) well explained the cat's behavioral responses function (Fig. 5E and F). It should be noted that the neurometric functions over shorter time scales showed a similar shape to that of the mean firing rate, suggesting that the spike time of the non-synchronized responses did not provide additional information to represent the click repetition rate.

### Percentage of synchronized response in the A1 population

**Figure 6:**
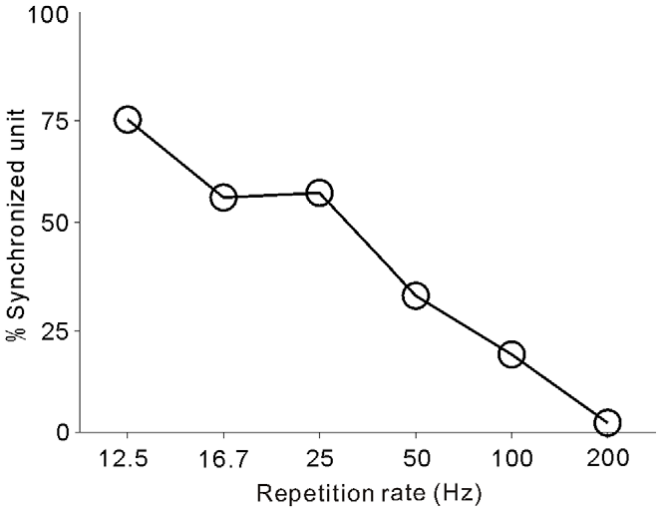
Percentage of synchronized units at the various click repetition rates.

As indicated by the above examples, the ability of synchronization to click train varied among the A1 neurons. Figure 6 summarizes the percentage of units showing significant synchrony (RS >13.8) to the clicks at various repetition rates. At the repetition rate of 12.5 Hz, 75% of 92 recorded units showed a synchronized response. The percentage of synchronized units slightly decreased to 58% when the repetition rate increased to 25 Hz, and then sharply decreased to 2% at the repetition rate of 200 Hz. This result is consistent with previous reports that most cortical neurons only synchronize to the repetition rate <50 Hz (Fig. 8 in[11] and Fig. 3d in [20]).

### Comparison of neurometric and psychometric functions in different neural populations

**Figure 7:**
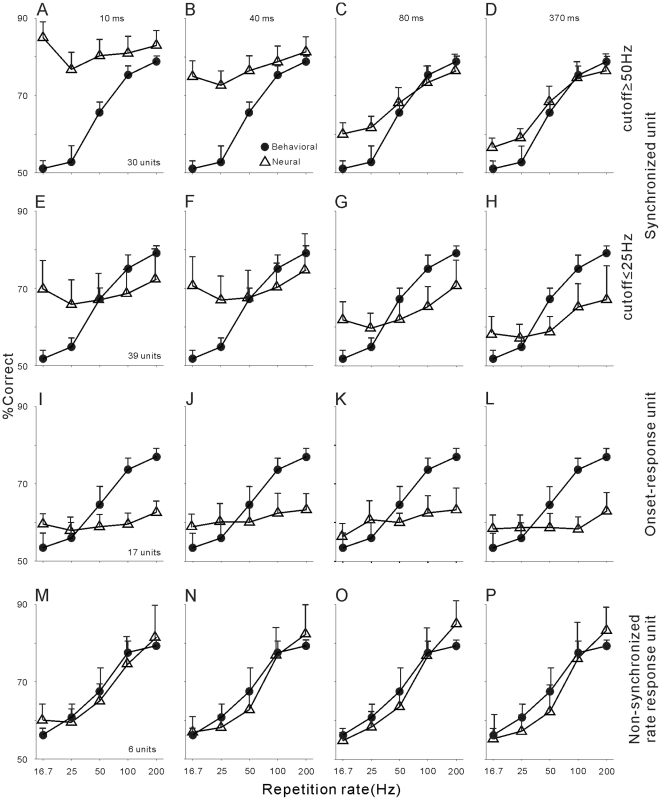
Comparison of the population neurometric and psychometric functions. (A)–(D): Triangles show the mean of neurometric functions averaged across 30 synchronized units with a synchrony cutoff ≥50 Hz in 10, 40, 80 and 370 ms time scales, respectively. Filled circles show the mean of psychometric functions obtained while recording these units. Horizontal bar represents 2SE. (E)–(H): Neurometric and psychometric functions of 39 synchronized units with a cutoff ≤25 Hz. (I)–(L): Neurometric and psychometric functions of 17 non-synchronized units in which the firing rate was not modified by the repetition rate of clicks. (M)–(P): Neurometric and psychometric functions of 6 non-synchronized units in which the firing rate was modified by the increase of the repetition rate.

According to whether the unit showed significant synchrony to 12.5 Hz clicks, we defined 69 “synchronized units” of the 92 recorded units. The synchronization ability of these units decreased with the increase to repetition rate. In our samples, we did not find a unit which did not synchronize to 12.5 Hz clicks, but synchronized to other higher repetition rates. Because the synchronized units may yield different types of neurometric functions, as shown by the examples in Figs. 2 and 3, we further divided the 69 synchronized units into two subgroups, according to whether the synchrony cutoff was ≤25 Hz or ≥50 Hz. Units with a higher synchrony cutoff generally showed a significant difference in the mean firing rate (p<0.001, ANOVA), while those with a lower synchrony cutoff did not. Figure 7A–D shows the mean and 2SE of the neurometric (triangles) and psychometric (filled circles) functions of 30 units with a synchrony cutoff of ≥50 Hz. Similar to the representative unit in Fig. 2, neurometric functions in 10–40 ms time scales (Fig. 7A and B) were obviously higher than psychometric functions at 16.7–50 Hz repetition rates, while those in 80–370 ms time scales well matched the psychometric function (Fig. 7C and D), because the firing rate of these units was modulated by the repetition rate. Hence, the cats may rely more on the firing rate of these units to perform the discrimination task.

The comparison of neurometric and psychometric functions in the 39 units of lower synchrony (cutoff ≤25 Hz) is shown in Fig. 7E–H. As represented by the unit in Fig. 3, the neurometric functions in any time scales did not well match the psychometric functions, suggesting that the spike activity of these units may not directly correlate with the cat's behavioral response.

The resting 23 units were classified into two groups. Six units showed a monotonic increase in the mean firing rate with the increase of the repetition rate, as shown in the example in Fig. 5. The change of the mean firing rate was statistically significant (p<0.01, ANOVA). These units were called as non-synchronized rate response units following the termination in the previous study of awake marmoset (Fig.2a in [20]). Seventeen units were termed as onset-response units, because they showed excitatory response at the stimulus onset of all tested click trains, as in the example in Fig. 4. Consequently, the mean firing rate was not significantly modified by the repetition rate. The comparison of neurometric and psychometric functions in the two types of units is shown in Fig. 7I–P. The onset-response units could not discriminate the click-trains in any time scales (Fig. 7I–L), even when the cat could perform the discrimination task well during the unit recording. In contrast, the non-synchronized rate response units discriminated the click-trains in parallel with the cat's behavioral response (Fig. 7M–P). The similarity among neurometric functions in different time scales suggests that the spike time of these units did not convey more information for discrimination of the repetition rate.

Taken together, the cat's behavioral response can be explained by the firing rate of “a part” of the A1 units. Because we only sparsely sampled a few cortical sites from each animal using the implanted electrodes, the result did not show a clear tendency in the spatial distribution of different cell types.

### Neurometric functions based on the pooled units

**Figure 8:**
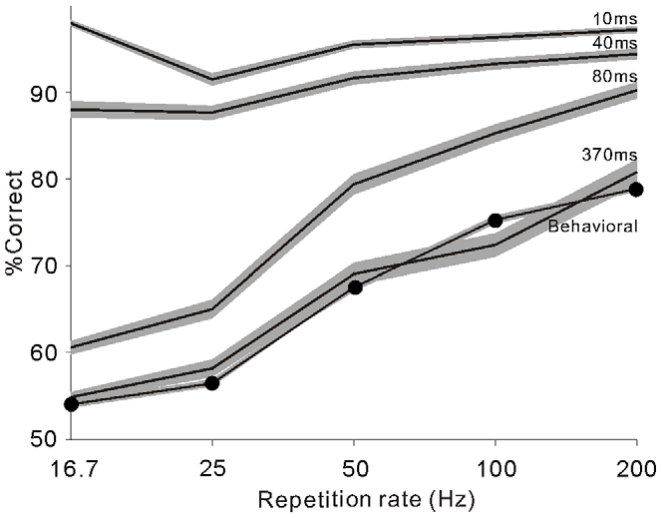
Comparison of the neurometric and psychometric functions obtained from the pooled data of 10 randomly selected units. Black line shows the mean of functions averaged across 100 repetitions of random selection. Shade area shows the 2SE. Circle marked line represents psychometric functions; others represent the neurometric functions in different time scales.

We then explored discrimination performance when observing output from multiple A1 units together. For this, we randomly selected 10 units from the 92 samples and constructed the neurometric function after pooling their spike activities. At the same time, the psychometric function was estimated by averaging the individual psychometric functions obtained during the recording of each unit. This procedure was repeated 100 times. The means of the 100 neurometric and psychometric functions are shown by the black lines and the line with circles in Fig. 8. The shaded area shows SE. It is clear that the neurometric function in short time scales outperformed the psychometric function, while the neurometric function of the mean firing rate well matched the psychometric function. This result further suggests that the neurons in the downstream stations of A1 may read out the firing rate information to direct the cat's behavioral response.

### A1 responses are not modulated by the cat's perceptual reports

**Figure 9:**
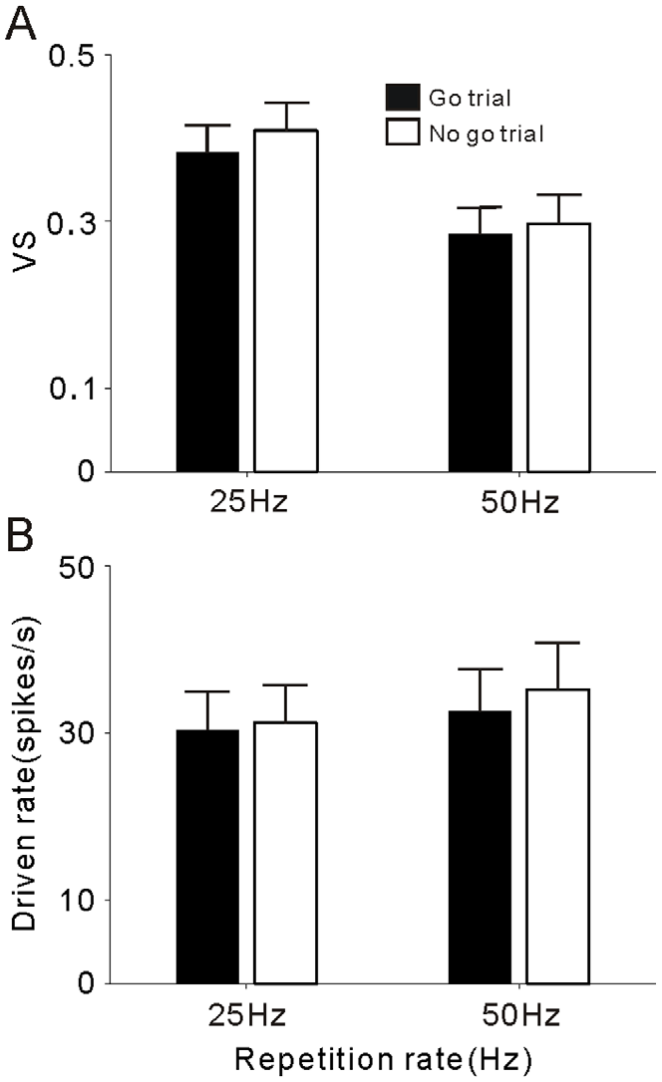
Comparison of neural population activity during Go and No-go trials for 25 and 50 Hz stimuli. (A) Black and white bars show the mean VS averaged over Go and No-go trials, respectively. Short horizontal lines mark 2SE. (B) The mean driven rate averaged over Go and No-go trials. No significant difference was found between the mean VS and driven rate of Go and No-go trials (t-test, p>0.05).

The other interesting issue is whether the activity of A1 neurons was affected by the ‘Go’ or ‘No-go’ judgments that the cats made on a trial-by-trial basis. To test this, we compared the neural responses during ‘Go’ and ‘No-go’ trials for 25 and 50 Hz stimuli, which are the near-threshold repetition rates for the discrimination task. We selected the 67 units recorded when the %Go responses of 25 and 50 Hz stimuli were between 25% and 75%, reflecting that the cat's judgment was ambiguous. The mean and 2SE of VS and driven rate (the firing rate of each unit was normalized by subtracting its background firing rate) of Go and No-go trials at 25 and 50 Hz repetition rates are shown in Fig. 9A and B. No significant difference (t-test, p>0.05) was found in the neural activities between Go and No-go trials, suggesting that the population activity of A1 did not affect the cat's judgment. Also, the comparison conducted on individual units did not find any units showing a significant difference in the VS and firing rate of Go and No-go trials.

## Discussion

In this study, we developed an important process to record spike activity from implanted electrodes in freely moving cats and investigated how the evoked neuronal activity in A1 can be used to explain the cat's performance during click-train discrimination.

### Comparison with previous results from passive listening animals

We found that 75% of A1 units exhibited a synchronized response to click-trains and the response synchrony decreased with the increase of the stimulus repetition rate (Fig. 6). The prevalence of synchronized response in A1 has been well reported in a number of previous studies on anesthetized animals [5], [6], [7], [8], [9], [10], [11], [12], [13], [14], [15], [16]. Studies on awake marmosets found a population of non-synchronized neurons tuned to the stimulus repetition rate, and proposed that the firing rate of non-synchronized neurons underlies the perception of pitch and flutter [18], [19], [26]. Non-synchronized neurons were also found in recent studies on awake macaques, but their fraction in A1 samples largely varied among different studies: 2% in Malone [12]; 17 % in Yin [21] and ∼50% in Lu [20]. This discrepancy has been attributed to the differences in many experimental factors, such as the stimulus properties, criteria to identify synchronization, the species, and biases in the sampling of neurons [21]. In this study, we also found 25 % of A1 units did not synchronize to any clicks at repetition rates of 12.5–200 Hz and 7% of our sample showed a monotonic increase of the mean firing rate with an increase of the repetition rate (Figs. 4 and 5). The general accordance between our and previous results indicates that the neural data recorded using our freely moving procedure were comparable with those collected using traditional methods.

### Spike time vs. firing rate

Co-existence of multiple response patterns may be useful for A1 to robustly encode temporal signals. An important question is how the neural codes are read out to direct behavioral responses. By comparing the cat's psychometric functions with the neurometric functions, we found that: 1) 30 units (33%) with higher synchrony ability (cutoff ≥50 Hz) could rely on spike time information to precisely discriminate all repetition rates, but the cats showed deteriorated behavioral performance at slow repetition rates (16.7–50 Hz), as predicted from the firing rate of these units (Figs. 3 and 7A–D). 2) 39 units (42%) with lower synchrony ability (cutoff ≤25 Hz) could also discriminate all the repetition rates depending on the spike time information, resulting in a neurometric function departing from the psychometric function. On the other hand, neural discrimination based on the firing rate was lower than behavioral discrimination, because the mean firing rate of these units was not reliably modulated by the repetition rate (Figs. 4 and 7E–H). 3) 17 onset-response units (18%) showed poor neural discrimination in any time scales, due to the similarity of firing patterns in response to all click-trains (Figs. 5 and 7I–L). 4) 6 non-synchronized rate response units (7%) showed a neurometric function matching the psychometric function in all time scales (Figs. 6 and 7M–P) because their firing rate was significantly modulated by the click repetition rate and the spike time did not correlate to the clicks. Moreover, the neurometric function based on the mean firing rate of 10 randomly selected units well matched the cat's psychometric function; therefore, the cats may read out the firing rate of A1 units to perform the behavioral task.

The roles of spike timing and firing rate in sensory neuroscience have been long debated. Previous studies in the auditory cortex showed that complex sounds such as communication vocalizations were represented by the precise spike timing information of A1 neurons [25], [27], [28]. For example, Walker et al. found that temporal discharge patterns of ferret's A1 neurons closely matched human psychometric performance, but only if the spike patterns were resolved in < 20 ms bins[28] Moreover, Engineer et al. showed that the neural discrimination based on time windows of 1–10 ms best predicted rat's discrimination ability of speech sounds [25]. One limitation of these studies is that they did not directly compare electrophysiological and psychophysical data from the same subject.

Comparison between neural and behavioral data has been well conducted in visual and somatosensory cortex. It has been indicated that firing rates averaged across 50–500 ms can be used to discriminate the sensory information of visual motion[29], [30], binocular depth[31], roughness of texture [32], [33], vibrotactile amplitude[34] and frequency[35], [36]. Among these, the data most comparable to ours are the neural activities of primary somatosensory cortex (S1) recorded from the monkeys performing a discrimination task of vibrotactile frequency[35], [36]. Most of the quickly adapting neurons of S1 showed phase-locked responses to the periodic mechanical sinusoids. However, some neurons also showed a rate-coding of stimulus frequency, with higher firing rates (averaged over the duration of a stimulus) in response to higher stimulus frequencies. The neurometric functions based on firing rate typically matched the psychometric functions, whereas neurometric functions based on the spike periodicity (spike timing information) predicted much more accurate performance than the monkey's actual behavior [35]. This result favored firing rate over spike timing as the neural code for perception of vibrotactile stimuli. Recently Lemus et al. conducted a similar experiment that recorded A1 neural responses while the monkeys performed a discrimination task of acoustic frequency [24]. The result also supports that firing rate, not spike timing, represents the neural code relevant for acoustic flutter discrimination.

In this study, we found that neural performance based on the precise temporal discharge pattern was good enough to perfectly distinguish all 16.7–200 Hz clicks from 12.5 Hz click; however, the cats in our experiment did not perform as well as predicted from spike timing. Their behavioral performance more correlated with the firing rate of A1 units. In other words, the timing of A1 spikes evoked by the click-train was very precise, but it was not exploited by the cortical circuitry, at least not to its full capacity. This result may be a consequence of the behavioral paradigms in our experiments. Before electrophysiological recording, our cats were over trained to show a Go response to a fast click-train (200 Hz) and a No-go response to a slow click-train (12.5 Hz). Because few A1 units (2% in our sample, Fig. 5) showed a synchronized response at a 200 Hz repetition rate, it is more convenient for the cats to use the difference in firing rates to discriminate 12.5 and 200 Hz repetition rates. During the recording session, a series of click-trains at various repetition rates were randomly presented, and only one click-train was presented per trial. In each trial, the cats had to compare the stimulus to the general concept of “slow” vs. “fast clicks” stored in the memory for correct discrimination. Because the original concept of slow and fast clicks may be established on the firing rate, the cats performed the task according to the firing rate; therefore, our result is good evidence indicating that rate-coding in the auditory cortex contributes to the behavioral response.

On the other hand, our results imply that the cats may have the potential to correctly discriminate the difference between 12.5 and 16.7 Hz click-trains, because discriminative information is well encoded by spike timing in A1. As mentioned above, if we had specially trained the cats to discriminate 16.7 Hz from 12.5 Hz click-train before the recording experiment, they might have achieved higher performance for the 12.5–16.7 Hz discrimination. Also, if the cats had been trained in an “AA” versus “AB” discrimination task (where A and B represent two different click repetition rates), their discriminability between the slow repetition rates might be improved. These possibilities are needed to be examined in future studies. Such studies will reveal the link between spike timing and auditory perception and clarify whether cortical information is processed in variable temporal precision according to the difference in behavioral requirements.

### Neural representation of behavioral meaning

In this study, we did not find a significant difference between the neural responses of A1 in Go and No-go trials of near-threshold stimuli (Fig. 9). Hence, the activity of A1 may not directly relate to the cat's judgment of the Go or No-go response. Instead, it may serve as input to an additional processing stage(s) to determine whether a click repetition rate is slow or fast. This is consistent with the study on monkeys, which suggests that A1 does not participate in the working memory or in decision-making in the frequency discrimination task [24]. Neurophysiologic observations in the primary somatosensory cortex (S1) also reported that the working memory and decision-making processes reside outside of S1 [37], [38]. Neural activity relevant to the behavioral meaning during auditory discrimination tasks has been found in the prefrontal cortex [39] and ventral premotor cortex [40]. To well understand the neural circuit underlying the cognitive process of auditory discrimination, the assigned functions of the early auditory cortices, such as the secondary auditory cortex, anterior auditory field and posterior auditory field, need to be further investigated with more elaborate paradigms in which both neuronal activity and psychophysical behavior are monitored and quantified simultaneously. In particular, the idea that neural representation of the repetition rate progressively changes to a monotonic rate code lacking stimulus-synchronized responses from A1 to non-A1 areas [19] is worthy of verification in a future study.

## Materials and Methods

All animal works was carried out in strict accordance with the recommendations in the Guide for the Care and Use of Laboratory Animals of the National Institutes of Health. The protocol was approved by the Committee on the Ethics of Animal Experiments of the University of Yamanashi (No.19–15). All surgery was performed under sodium pentobarbital anesthesia, and all efforts were made to minimize suffering.

### Psychophysical testing procedure

Using a method similar to our previous studies [41], [42], [43], we trained 7 cats to perform a Go/No-go task to discriminate different acoustic stimuli. Initially, the cats were deprived of food to 80% of their free-feeding body weight, but had free access to water. Subjects were tested in a custom-built behavioral cage that was acoustically transparent and placed in an electrically shielded, sound-attenuated chamber. Training was computer-controlled using custom-built software in a MATLAB (Mathworks) environment that interacted with the apparatus via digital input–output hardware (PCI-6052E; National Instruments). Auditory stimuli were also digitally generated by the custom-built software and delivered via a pair of speakers (K701; AKG) placed outside the grid walls of the behavior box. Sound calibration was conducted using a Bruel & Kjaer 1/2″ condenser microphone with a preamplifier 2669 situated at the cat's ear. Sound pressure level (SPL) is expressed in decibels relative to 20 µPa. The system frequency transfer function was flat up to 32 kHz (±6 dB). A video camera and photoelectric sensors were used to monitor the cat's position and movement. The cats were first trained to lick a metal pipe on sound presentation to obtain a drop of liquid food. Each trial was initialized only when the cats stood ready and kept their head in the observing position in front of the metal pipe, where the speaker calibration was performed. Cats learned this procedure within one week and were then trained to distinguish two click-trains (monopolar, rectangular pulse of 0.2 ms duration; 320 ms train duration) with a repetition rate of 12.5 and 200 Hz, respectively. Stimulus amplitudes were adjusted to the subjective intensity equal to 60 dB SPL of 4 kHz pure tone. We defined a 200 Hz click-train (5 ms in inter-click-interval) as the target and a 12.5 Hz click-train (80 ms inter-click-interval) as the standard. Subjects were required to lick the metal pipe when a target was presented (hit), and not to lick when a standard was presented (correct rejection). There were also two kinds of error responses: licking when a standard was presented (false alarm) and not licking at the presentation of a target (miss). Subjects were positively reinforced only for the hit response. In each 100-trial session, the trials of target and standard stimuli were randomly presented in an equal ratio.

The subjects' daily performance in this task was quantified via measure d' from signal detection theory [44], [45]. The *d'* is calculated as *d'*  =  Z(hit-rate) - Z(false-alarm-rate), where Z represents z-transform of the probability of hit and false alarm responses. Z-transform calculates the inverse of the normal cumulative distribution with a mean of 0 and standard deviation of 1. The cats required about one month of training (3,000–4,000 trials) to establish a stable performance (*d’* ≥2.0). We then tested how the cats respond to the click-trains at various repetition rates, by randomly presenting 50 trials of 12.5 Hz click-trains as standard and 10 trials of 200, 100, 50, 25 and 16.7 Hz click-trains as a target. It should be mentioned that the actual stimulus duration of 16.7 Hz click-train was 300 ms, because its inter-click interval of 60 ms is not an exact divisor of 320 ms.

### Surgical preparation and electrode implantation

The cats were anesthetized by sodium pentobarbital (30 mg/kg) and fixed to a stereotaxic frame (SN-3N; Narishige). The position of A1 was marked on the bone surface according to stereotaxic coordinates. Four small holes were drilled over the occipital bone and fine jeweler's screws were inserted to serve as an anchor for a metal block that was cemented to the skull with dental acrylic. After the cement had hardened, the head was held through the metal block and the ear bars were removed. We then drilled several small holes (0.5–1 mm diameter) in the temporal bone above the potential location of A1. A tungsten microelectrode (diameter: 250 µm; impedance: 2–5 MΩ at 1 kHz; FHC Inc.) was advanced into the cortex using a micromanipulator to examine the neural responses to tonal stimuli at each site. According to the characteristics of the tonotopic gradient, we identified the location of A1. We then implanted a microwire array following the method developed by Jackson and Fetz [46]. The microwire consisted of 12 (2×6) Teflon-insulated 50-µm diameter tungsten wires (part #795500; A–M Systems, Carlsborg, WA) running inside polyamide guide tubes of 225-µm internal diameter (part #822200; A–M Systems). The tip impedance of each wire was around 0.5 MΩ at 1 kHz. For implantation, a 5×3 mm craniotomy was made at the location of A1 with a dental bur. The microwire array was then lowered into position using a costume-made manipulator so that the ends of the guide tubes rested just above the dura mater over the low frequency (<16 kHz) area of A1. Wires were inserted into the cortex until the tips of the electrodes were 1.0–2.0 mm below the dura, while viewing through a microscope and listening to an audio monitor of the recorded signal. Then, the craniotomy was filled with SILASTIC, a silicone elastomer (World Precision Instruments) and sealed using dental acrylic. Plastic casing was attached with further skull screws and cement.

### Electrophysiological recording

The recording experiment started after 1–2 weeks of postoperative recovery. The physiological recording was conducted while the free-moving cats were responding to the random presentation of various click-trains (test session) in the behavioral box. The microwire output was connected to a multi-channel preamplier (RA16PA; TDT, Alachua, FL, USA) using a flexible, low noise cable. The output of the preamplifier was delivered to a digital signal processing module (RX-7; TDT). Spike activities were discriminated using principal component feature space spike-sorting software (SpikePac; TDT). Each day we recorded one 100-trial test session, which was started after the cats had practiced 100–200 trials as a training session. We selected the data recorded in the session with the best behavioral performance (the lowest false alarm rate and the highest hit rate) for further analysis. After completing the record in one hemisphere, the implantation surgery and recording procedure were repeated on the other hemisphere.

At the end of the experiment, the animal was deeply anesthetized and perfused with 10% formalin. The cerebral cortex was cut into coronal sections and stained with neutral red. The recording sites were confirmed according to the lesions caused by the electrode tips. This report was based on the units from A1.

### Data analysis

Spike activities driven by click-trains were aligned along the stimulus onset, constructing a raster plot of each repetition rate (Fig. 2A). The peri-stimulus time histogram (PSTH), counting the spikes across different repetition rate, was computed in 1-ms bin width (Fig. 2B, for visualization purpose, the PSTH was smoothed by Gaussian function with 5 ms SD). The mean firing rate was calculated over the time period from stimulus onset to 50 ms after stimulus offset. Vector strength (VS) [47] was used to measure the degree to which the neural response was concentrated in a particular phase of the repetition period of the clicks, such that 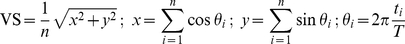
where n is the total number of spikes, t_i_ is the time of spike occurrence, and T is the inter-click interval. VS values ranged from zero (spikes evenly distributed throughout the stimulus period) to one (spikes are perfectly aligned to a particular phase of the stimulus period). The VS of each neuron was calculated over the time period starting from 50 ms after stimulus onset to 50 ms after stimulus offset. A neural response was considered to be synchronized to the click if the Rayleigh statistic (RS), 2 nVS^2^, exceeded 13.8 (p <0.001) [48].

We used the Spike Distance Metric (SDM) [42], [49], [50], [51] to test whether an ideal observer can discriminate the repetition rate based on the neural responses. For this, spike trains generated by each unit in response to each sound presentation were converted into a PSTH (10 ms bin; time window: from stimulus onset to 50 ms after stimulus offset). The PSTH was normalized by the maximum bin height of all PSTHs obtained from the same unit. A spike distance between a pair of PSTHs was computed as the Euclidean distance (ED), which is a square root of the sum of squared differences between firing rates at each bin(i).


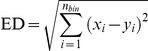
, where n_bin_ is the total number of bins, and x and y are bin heights.

We then used a classification scheme based on the spike distance metric (SDM) to quantify the neural discrimination of the temporal interval. Ten trials of PSTH recorded during the presentation of standard stimulus (12.5 Hz click-train) were randomly selected and compared with the 10 trials of each target stimulus (16.7, 25, 50, 100 or 200 Hz click-train) for this analysis. Firstly, a pair of PSTHs was randomly chosen for the standard and target stimuli, respectively. The remaining PSTHs were then assigned to a standard or target stimulus, depending on which template was closest based on the ED measure. This procedure was repeated 100 times for different pairs of template in each standard-target comparison (12.5–16.7, 12.5–25, 12.5–50, 12.5–100, and 12.5–200 Hz, respectively).

The percentage of correct classification (% correct) was used as a measure of discrimination. The chance level for classification was 50 %, because a PSTH could be equally assigned to the standard or target interval when there were no response preferences. If neurons with a high ability of repetition rate discrimination showed specific temporal response patterns corresponding to a specific click-train, making a small within-category ED and a large across-category ED, the PSTHs were more frequently assigned to the template with a small ED, resulting in a higher % correct. A neurometric function for each unit was constructed by plotting % correct against the click repetition rate (thin line in Fig. 2D). We also constructed PSTHs using bin sizes of 40, 80 and 370 ms, respectively. A larger bin size will capture more firing rate information, while a smaller bin size will preserve more spike time information. SDM analysis was conducted on PSTHs of various bin sizes to explore the temporal precision of neural encoding.
